# Changing patterns of infectious diseases in children during the COVID-19 pandemic

**DOI:** 10.3389/fcimb.2023.1200617

**Published:** 2023-06-29

**Authors:** Ming-Chun Yang, Yu-Tsun Su, Ping-Hong Chen, Ching-Chung Tsai, Ting-I Lin, Jiunn-Ren Wu

**Affiliations:** ^1^ Department of Pediatrics, E-DA Hospital, I-Shou University, Kaohsiung, Taiwan; ^2^ School of Medicine, College of Medicine, I-Shou University, Kaohsiung, Taiwan; ^3^ School of Medicine for International Students, College of Medicine, I-Shou University, Kaohsiung, Taiwan

**Keywords:** coronavirus disease 2019, immune debt, influenza, pediatric, pneumococcus, respiratory tract infection, respiratory syncytial virus

## Abstract

Each infectious disease has had its own epidemic pattern and seasonality for decades. However, public health mitigation measures during the coronavirus disease 2019 (COVID-19) pandemic have resulted in changing epidemic patterns of infectious diseases. Stringent measures resulted in low incidences of various infectious diseases during the outbreak of COVID-19, including influenza, respiratory syncytial virus, pneumococcus, enterovirus, and parainfluenza. Owing to the prevalence of severe acute respiratory syndrome coronavirus 2 (SARS-CoV-2) infections and subsequent immunity development, decreasing virulence of SARS-CoV-2, and worldwide immunization against SARS-CoV-2 in children beyond 6 months of age, mitigation measures are lifted country by country. Consequently, the immunity debt to infectious respiratory viruses other than SARS-CoV-2 contributed to the “off-season,” “see-saw,” and “upsurge” patterns of various infectious diseases in children. Moreover, apart from the persistence of SARS-CoV-2, the coexistence of other circulating viruses or bacterial outbreaks may lead to twindemics or tripledemics during the following years. Therefore, it is necessary to maintain hand hygiene and immunization policies against various pathogens to alleviate the ongoing impact of infectious diseases on children.

## 1 Introduction

The coronavirus disease 2019 (COVID-19) pandemic has increased worldwide since the beginning of 2020, resulting in devastating medical and economic impacts. Globally, countries have adopted various response strategies to slow the spread of COVID-19 (Su et al., 2021). These mitigation measures not only influenced transmission of severe acute respiratory syndrome coronavirus 2 (SARS-CoV-2), but also resulted in changing patterns of infectious diseases, especially those transmitted by respiratory pathogens. Since the beginning of the COVID-19 pandemic, severity of COVID-19-related illness, mutation of SARS-CoV-2 variants, and prevalence of other respiratory viruses and their relevant diseases change continuously. Here, we synthesized the current knowledge on epidemiological features regarding the trends of SARS-CoV-2 infection and other infectious diseases in children during the COVID-19 pandemic.

## 2 SARS-CoV-2 infection and reinfection in children

### 2.1 Changing severity in children with SARS-CoV-2 infection

Although the COVID-19-related symptoms and prognosis are better in children than in adults (Ludvigsson, 2020), COVID-19 has an unneglectable impact on children. The major SARS-CoV-2 variants evolved from pre-Alpha, Alpha, and Delta, to Omicron (Mattiuzzi and Lippi, 2022). The hospital admission rate in children with COVID-19 varies widely and is influenced by the evolving SARS-CoV-2 dominant variants, healthcare system availability, and different age groups and immunization status. At the beginning of the COVID-19 pandemic in 2020, multinational studies from Europe and China revealed that 51–62% of children required hospital admission, 7–8% required intensive care unit (ICU) admission, and 4% required mechanical ventilation (Götzinger et al., 2020; Bhuiyan et al., 2021). Clinical severity in children has considerably changed over time, after the initiation of immunization programs and evolution of SARS-CoV-2 variants. By 2021, most children had mild symptoms (58%) or were asymptomatic (36%) (Say et al., 2021). Following the extension of immunization to infants aged >6 months, hospitalization rates declined to 0.02–0.2% in fully vaccinated children (González et al., 2022; Tan et al., 2022). Thereafter, hospitalization rates stabilized and remain low to date.

### 2.2 SARS-CoV-2 reinfection in children

Children who recovered from COVID-19 generated a robust immune response and developed protective immunity. A meta-analysis concluded an 87% reduction in SARS-CoV-2 reinfection in those previously infected compared to those who were never infected (Deng et al., 2022). Moreover, even though children and adolescents were either asymptomatic or experienced only mild symptoms during their first SARS-CoV-2 infections, all participants demonstrated seropositive SARS-CoV-2-specific antibodies. Although the antibody titer in these children was low compared to that in adults, 92% still exhibited virus-neutralizing activity, which may protect them from reinfections (Szépfalusi et al., 2021). Interestingly, SARS-CoV-2 antibody titers did not differ between asymptomatic and symptomatic children (Maier et al., 2022). In addition to humoral immunity, IgA antibodies play a role in mucosal immunity by eliminating viral replication and reducing the risk of reinfection (Zohar and Alter, 2020). Asymptomatic infections are more common in children (Yasuhara et al., 2020), and the trend of receiving SARS-CoV-2 testing is gradually decreasing (Park et al., 2023). As of August 2022, a national commercial laboratory seroprevalence study demonstrated that approximately 86% of children and adolescents in the US had serologic evidence of previous SARS-CoV-2 infection (Son et al., 2023).

The risk and picture of SARS-CoV-2 reinfection are changing, and the emergence of new variants can partially evade immunity from previous infection or vaccination (Milne et al., 2021). The risk of reinfection increases with age in infants, children, and adolescents (Mensah et al., 2022; Medić et al., 2023). A previous study reported a J-shaped curve of antibody titers with respect to age after the first SARS-CoV-2 infection. Antibody titers were lowest among adolescents and young adults, higher in younger children, and highest among older adults (Maier et al., 2022). The J-shaped distribution of antibodies may partly explain the high risk of reinfection in adolescents.

Fortunately, the risk of symptomatic COVID-19 reinfection was lower in seropositive children, whose relative risk of being symptomatic was reduced to 36–51% (Kubale et al., 2022). Although moderate and severe cases could still occur in reinfection, there was no difference in severity between first and second infections (Kubale et al., 2022). Therefore, recurrent infection was not a risk factor for severe COVID-19.

## 3 "Declines" in infectious diseases in children during the early COVID-19 pandemic

### 3.1 Declines in RSV, influenza, and other viral infections

During the first wave of the COVID-19 pandemic, public health measures were strictly applied, including social distancing, increased awareness of wearing masks, reinforced hand hygiene, reduced contact between children, maintaining ventilation, containment, curfew, and closure of schools and daycare centers. Implementation of public health measures led to abrupt declines in human respiratory syncytial virus (RSV) and influenza infections worldwide in 2020 and 2021 due to a reduction in the circulation of respiratory viruses, with a reported reduction in infection rates of up to 98–99% (Baker et al., 2020; Feng et al., 2021; Stamm et al., 2021; Stera et al., 2021; van Summeren et al., 2021; Yeoh et al., 2021; Binns et al., 2022; Zhang et al., 2023). Declines in RSV and influenza and reduced transmission of other respiratory viral pathogens, including common human coronavirus, parainfluenza viruses, human metapneumovirus, adenovirus, rhinovirus, and enterovirus have been observed in the US (Olsen et al., 2021; Rankin et al., 2023).

Bruggink et al. also reported a significant reduction in enterovirus infections during the 2020 early COVID-19 pandemic period in Australia. Mitigation measures resulted in an 84.2% reduction in enterovirus specimen positivity rate compared to previous decades, as the number of tested specimens did not differ appreciably in 2020 from that during 2010–2019 (Bruggink et al., 2021).

### 3.2 Declines in pneumococcal and other bacterial infections

The incidence of invasive diseases due to *Streptococcus pneumoniae*, *Haemophilus influenzae*, and *Neisseria meningitidis* in early 2020 was significantly reduced in 26 countries (Brueggemann et al., 2021). Furthermore, the number of invasive pneumococcal disease (IPD) cases in children in Spain in 2020 decreased by 65% compared to 2018–2019 (Ciruela et al., 2022). Similarly, pneumococcal infections among children in China showed a decreasing trend during the 2020 early COVID-19 pandemic period (Li et al., 2022). Interestingly, studies from Israel, France, and Belgium reported that even though pneumococcal infection reduced strongly in 2020 and early 2021, pneumococcal carriage in young children declined only slightly during the pandemic (Willen et al., 2021; Danino et al., 2022; Rybak et al., 2022). Therefore, the major explanation for the reduced pneumococcal disease was the full suppression of co-pathogens that can lead to secondary pneumococcal infections, including RSV, influenza viruses, and human metapneumovirus, instead of the decreased number of pneumococcal carriages (Willen et al., 2021; Danino et al., 2022; Rybak et al., 2022). A reduction in *Haemophilus influenzae* infections in children has also been reported in China. The positivity rates of *H. influenzae* infection in children with respiratory tract infections in 2020 (6.21%) and 2021 (7.37%) were lower than those in 2018 (11.28%) and 2019 (10.16%) (Zhou et al., 2023).

### 3.3 "Declines" in other infectious disease

An epidemiological and observational study in Northern Italy revealed a reduction in the prevalence of chickenpox, scarlet fever, pertussis, mumps, rubella, and measles in children (Belingheri et al., 2021). A report from the European Centre for Disease Prevention and Control also demonstrated that the number of measles cases declined in European Union countries and the United Kingdom in 2020 (Nicolay et al., 2020). Moreover, data from the China Disease Prevention and Control Information System showed a significant decrease in the number of varicella, measles, and rubella infections in 2020 (Wu et al., 2020).

Furthermore, the annual number of community-acquired infectious diseases among children in France decreased by one-third in 2020 compared to 2018 and 2019, including scarlet fever, acute tonsillopharyngitis, enteroviral infections, acute bronchiolitis, and gastroenteritis (Cohen et al., 2022). The incidence of acute otitis media, acute bronchiolitis, and croup also decreased among children in the US during the 2020 COVID-19 pandemic (Kaur et al., 2021).

The volume of pediatric emergency department visits and admissions also declined throughout the COVID-19 pandemic (DeLaroche et al., 2021; Moynihan et al., 2021; Miron et al., 2022b). A comprehensive systematic review further documented a 37% reduction in overall healthcare services, encompassing a 42% and 28% decrease in visits and admissions, respectively (Moynihan et al., 2021).

### 3.4 Declines in infectious diseases in Taiwan

**Figure 1:**
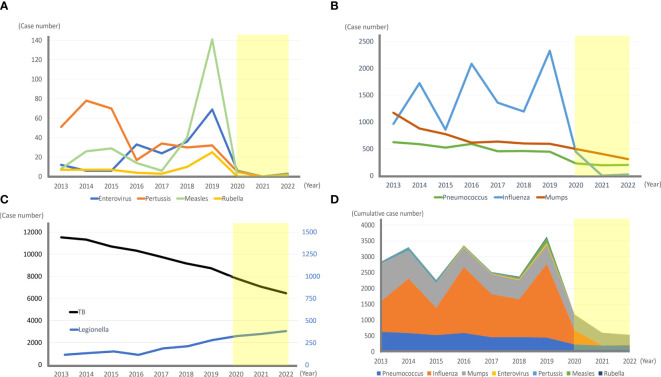
The incidence of various infectious diseases in Taiwan before (2013–2019) and during (2020–2022, light yellow box) the COVID-19 pandemic. **(A, B)** Annual reported cases of invasive pneumococcal diseases, influenza-associated critical illnesses, mumps, enterovirus-associated critical illnesses, pertussis, measles, and rubella in Taiwan from 2013–2022 are shown. The annual incidence dropped dramatically in 2020–2022 during the COVID-19 pandemic. **(C)** Annual tuberculosis cases decreased at a steady rate during the past 10 years, and the slope of 2020–2022 did not differ from the slope of 2013–2019 significantly. In contrast, the number of Legionella cases has steadily increased over the past 10 years. **(D)** Annual cumulative number of invasive pneumococcal diseases, influenza-associated critical illnesses, mumps, enterovirus-associated critical illnesses, pertussis, measles, and rubella significantly decreased from 2020 to 2022.

Country-based mitigation measures in Taiwan successfully delayed the COVID-19 epidemic until May 2022. Implementing mitigation measures also contributed to a decrease in the burden of other infectious diseases to hospitals. A report from Taiwan suggested an approximately 50% reduction in pediatric patient volume per hour at the emergency department between February 2020 and January 2021 compared with the pre-COVID-19 period (February 2019 to January 2020). The most obvious drop was noted for infectious diseases, especially influenza and enterovirus infections (Huang et al., 2022). The general population of Taiwan is approximately 23 million. According to the Taiwan CDC report (**Figure 1**), there was a 95–98% reduction in the rate of influenza-related critical illnesses in 2021–2022 compared to 2013–2019. The incidence of IPD, pertussis, enterovirus-related critical illness, measles, mumps, and rubella also decreased significantly during the COVID-19 pandemic. However, the tuberculosis incidence has declined annually since 2013 with a steady rate of de-escalation of 1,000 cases every two years, from 11,528 cases in 2013 to 6,387 in 2022; this change rate did not differ before and after the COVID-19 pandemic. Conversely, there was no decline in *Legionella pneumonia* infection rates, which steadily increased over the past decade, from 115 cases in 2013 to 382 in 2022 (Taiwan Centers for Disease).

## 4 Immune debt and resurgence of infectious diseases during the current COVID-19 pandemic

The lack of exposure to various infectious pathogens in children during the COVID-19 pandemic lockdown has an impact on their developing immunity. The paucity of protective immunity raises concerns regarding susceptibility to infectious diseases, the so-called immunity debt. Previously, an immunity debt was observed during the 2009 H1N1 influenza pandemic on RSV seasonality. Delayed onset of the RSV epidemic occurred initially, followed by a subsequent surge in the following year (Li et al., 2021).

### 4.1 “Off-season,” “see-sawing,” and “upsurge” patterns of RSV infections in children

**Figure 2:**
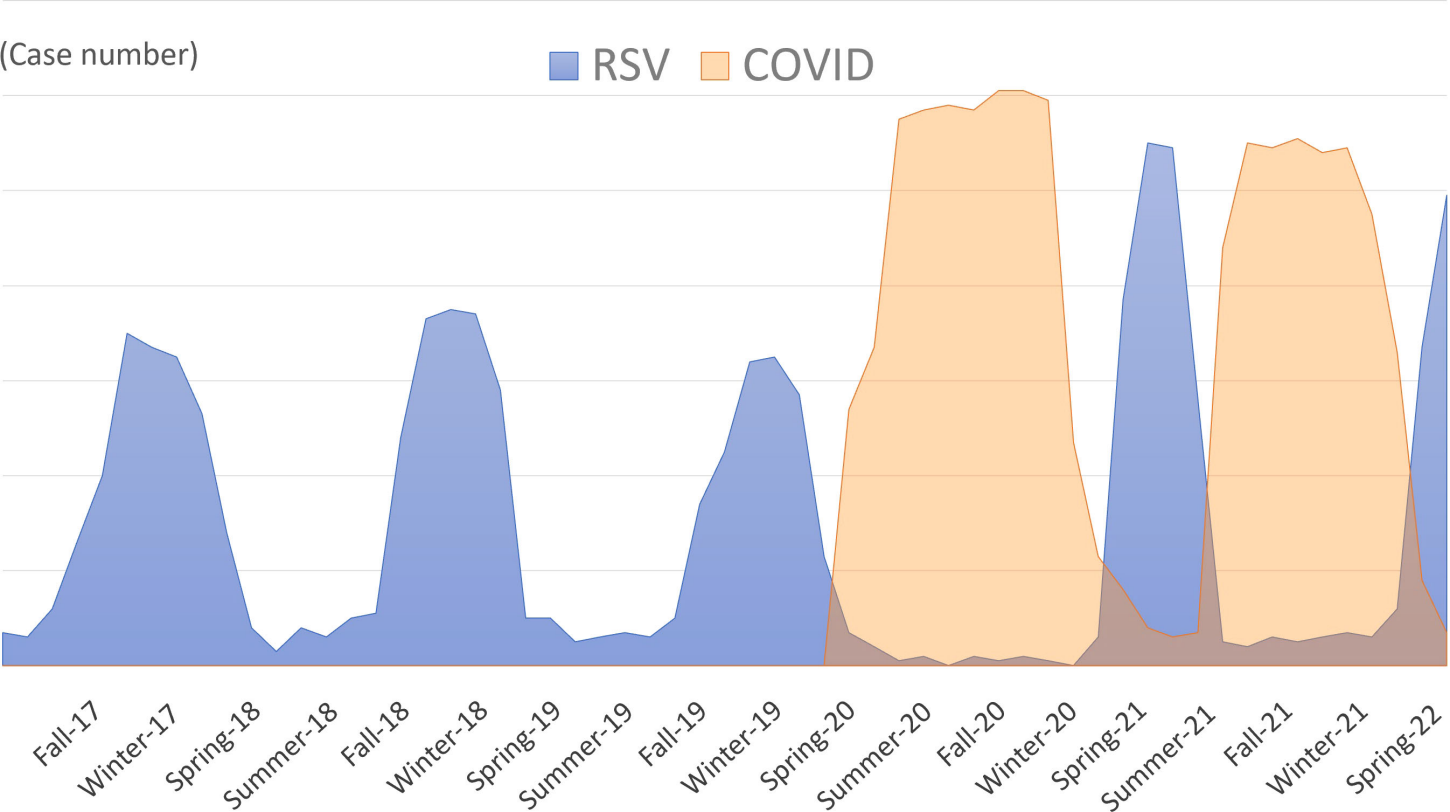
The schematic diagram showing the “off-season”, “see-sawing”, and “upsurge” patterns of respiratory syncytial viruses (RSV) infection in children. Previously RSV infections had a seasonal peak; however, the impact of COVID-19 mitigation measures contributed to the “off-season” resurgence of RSV. The “see-sawing” pattern of the RSV outbreak alternated with the COVID-19 epidemic. Strict restrictions during the COVID-19 outbreak periods result in the flattening of the RSV epidemic; in contrast, unrestrained regulations after the cooldown of COVID-19 led to resurgence of the RSV outbreak. Public health measures lead to immunity debt to RSV, which further results in RSV infections reaching the highest peak after the dismissal of public health mitigation policies (“upsurge” pattern).

Immunity debt leading to off-season resurgence of respiratory viral infections in several countries has been observed since 2021, after the wide-scale implementation of SARS-CoV-2 vaccination campaigns and easing of restrictive measures. Infants and children spent more time in daycare centers and schools than they did during the initial COVID-19 period; therefore, young children had a higher risk of infection. Previously RSV infections had a seasonal peak; however, the impact of COVID-19 mitigation measures contributed to “off-season” RSV bronchiolitis resurgence in France, England, and Israel (Casalegno et al., 2021; Delestrain et al., 2021; Rybak et al., 2021; Weinberger Opek et al., 2021; Billard and Bont, 2023). Furthermore, an alternating “see-sawing” pattern of COVID-19 and RSV infections in children has been observed in France, Italy, the US, and Australia (Agha and Avner, 2021; Delestrain et al., 2021; Eden et al., 2022; Rabbone et al., 2022). Strict restrictions during the COVID-19 outbreak periods flattened the RSV bronchiolitis epidemic; in contrast, unrestrained regulations after the cooldown of COVID-19 led to a resurgence of RSV infections (**Figure 2**).

Other than the “off-season” and “see-sawing” features, “upsurge” pattern occurs as well in respiratory infectious diseases during the COVID-19 pandemic (**Figure 2**). Several countries reported that RSV infection reached its highest peak after dismissal of public health mitigation policies. In Japan, the largest increase in RSV bronchiolitis cases in children began in 2021. The cumulative number of RSV infections rose from 570 in 2020 to 10,327 in the early-to-middle 2021 (Ujiie et al., 2021). In New Zealand, a rapid increase in RSV bronchiolitis in children aged 0–4 years was observed after relaxation of the strict border closure policy in April 2021. The RSV incidence was three times higher than in 2015–2019, which contributed to more hospitalizations and ICU admissions due to RSV in this age group (Hatter et al., 2021). The RSV upsurge in Australia occurred from late September to November 2020, when RSV infections exceeded the median 2012–2019 seasonal peak. Considering the prior waning of population immunity to RSV from 2019 to mid-2020, there were more RSV-naïve older children. Thus, the median patient age in 2020 was 18.4 months, which was significantly higher than the upper range between 2012–2019 (7.3–12.5 months) (Foley et al., 2021). Because of the delayed first RSV infection during the COVID-19 pandemic, RSV-related hospital admissions were higher among children aged 24–59 months compared to younger ages (Nygaard et al., 2023; Rao et al., 2023a).

A national population register in Norway revealed a surge in respiratory tract infection admissions from September to October 2021 among all children aged 0–5 years, which exceeded the numbers usually observed at the typical season peak in January. The major causes were RSV-related and lower respiratory tract infection-related admissions. Conversely, the number of influenza-related admissions remained low during this period (Methi et al., 2021). A study in Italy demonstrated an extensive epidemic of pediatric acute respiratory infections from June to October 2021. Compared to October 2019, RSV, parainfluenza, rhinoviruses/enteroviruses, metapneumoviruses, common coronaviruses, and adenoviruses were more frequently isolated from specimens in October 2021 (Mattana et al., 2022). It is worth noting that coinfections with two and three viruses were found in approximately 30% and 7% of all positive cases, respectively. This finding highlights the increasing circulation of these viruses by 2021 (Mattana et al., 2022).

### 4.2 Surge in influenza infections in children

The “see-sawing” and “upsurging” patterns were also observed for influenza infections. Following two years of decline in influenza virus circulation during the early COVID-19 pandemic, influenza infection rates increased in 2022, coinciding with a decrease in SARS-CoV-2 infections (Miron et al., 2022a; Lei et al., 2023; Rao et al., 2023b). Relaxation of non-pharmacological interventions subsequently resulted in a significant accumulation of influenza infections, leading to a widespread influenza outbreak.

### 4.3 Surge in IPD in children

The upsurge in infections after the COVID-19 pandemic also occurred in children with IPD. In a national enhanced surveillance in England, the children’s IPD incidence declined by 50% in 2020 but increased in 2021 after lifting COVID-19 social restrictions. Additionally, the 2021 children’s IPD incidence (1.96/100,000) was higher than that during the same period in pre-pandemic years 2017–2019 (1.43/100,000) (Bertran et al., 2022). A study in Germany also indicated that the children’s IPD incidence exceeded seasonal levels in 2021, concurrent with the dismissal of non-pharmaceutical interventions intended to lower the SARS-CoV-2 transmission. From April to June 2021, IPD among children exceeded 9% of the average monthly values for 2015–2019 (Perniciaro et al., 2022). Although large-scale IPD has not been reported in other countries, it is still necessary to pay attention to the surge after loosening public mitigation restrictions.

### 4.4 Surge in enterovirus infection in children

A similar upsurge was observed for enterovirus infections. Since there was a very low prevalence of enterovirus infection after hygiene reinforcement in France in 2020, reduced immune stimulation and greater susceptibility to enterovirus led to a large-scale outbreak of hand, foot, and mouth diseases (HFMDs) in children. A total of 3,403 cases were reported from January to September 2021, which was 47% higher than during the same period in 2018–2019 (Mirand et al., 2021). Likewise, after easing COVID-19 mitigation measures, enterovirus cases re-emerged in 18 European countries between January and October 2021. Although the number of samples tested monthly remained unchanged, the proportion of samples identified as enteroviruses increased from 2.5% in January to 8.2% in September. The positivity rate of enterovirus-D68 also increased from 0.2% in January to 14% in September 2021 (Benschop et al., 2021). Furthermore, soon after dismissal of non-pharmaceutical interventions in 2021, Brazil’s Information System for Notifiable Diseases reported a large-scale outbreak of HFMDs that was exclusively associated with the re-emergence of the Coxsackievirus A6 sublineage (Carmona et al., 2022). These outbreaks could be attributed to immunity debt to enteroviruses during the COVID-19 pandemic.

## 5 Discussion

The changing patterns of SARS-CoV-2, RSV, and influenza infections continuously evolve. It is worth noting that a tripledemic (COVID-19, RSV, and influenza) may occur following a low prevalence of RSV and influenza infections. Extensive adoption of molecular diagnostic tests, such as rapid multiplex PCR, has facilitated precise concurrent detection of various viral pathogens, offering potential advantages, including expedited patient discharge, targeted administration of antimicrobials, and efficient utilization of isolation facilities (Clark et al., 2023).

However, patients may not afford such accurate diagnostic investigations for economic reasons (Kelleni, 2023). Even in countries with sufficient healthcare systems, a shortage of medical resources may also occur. The rates of RSV- and influenza-associated hospitalizations of infants and children in late-2022 were higher than those during the same period in 2010-2020 (Centers for Disease Control and Infection, 2022). The emerging tripledemic has led to shortages in pediatric inpatient capacity, drugs, and equipment in the US in late-2022 (Furlow, 2023).

Fortunately, there are only a few reports on this tripledemic. Opinions have been proposed against the ongoing occurrence of a tripledemic. Based on the theory of viral interference, immune responses and virus-virus interactions at the host level may result in synergistic or antagonistic interactions. Fage et al. demonstrated that preceding infection with SARS-CoV-2 subsequently reduces the replication of influenza A (H1N1) and RSV in the human nasal airway epithelium. Furthermore, prior infection of the human nasal airway epithelium with influenza A (H1N1) also resulted in decreased SARS-CoV-2 viral load (Fage et al., 2022). Previous epidemiological surveillance revealed that RSV was detected less frequently during influenza epidemics (Anestad et al., 2007; Gröndahl et al., 2014; Meningher et al., 2014; van Asten et al., 2016). Chan et al. (2018) reported that infection of ferrets with influenza A (H1N1) induced a higher production of cytokines and immune mediators in the respiratory tract compared to RSV. Primary infection with influenza A (H1N1) prevented infection with RSV in ferrets, when infections were separated by seven days. However, both viruses induced minimal cross-reactive interferon-γ-producing cells, indicating that the reduced RSV infection was not caused by cross-reactive immunity between H1N1 and RSV. This suggests that innate immune mechanisms may be involved in the interference between H1N1 and RSV (Chan et al., 2018). Studies have also shown that sequential infection of human bronchial epithelial cells with human rhinovirus (48–72 h ahead) accelerates interferon-stimulated gene responses, which further impair SARS-CoV-2 replication (Cheemarla et al., 2021; Dee et al., 2021). Thus, the extent of the tripledemic might be limited by viral interference between these respiratory viruses. Although it is not necessary to panic, close attention should be paid to future viral dynamics. One way to prevent medical resources exhaustion is popularizing SARS-CoV-2 and influenza immunizations.

Since April 2022, there have been widespread reports of severe cases of acute hepatitis of unknown etiology in children (Namakin et al., 2023). It is noteworthy that the affected children exhibited a potential immunological predisposition. The disruption of typical patterns of exposure and immunity resulting from population lockdowns enforced during the COVID-19 pandemic may have rendered these children susceptible to viral coinfections (Matthews et al., 2022). Consequently, it is imperative to establish international infectious disease monitoring and foster collaborative efforts in the post-COVID-19 period.

The ever-changing patterns of infectious diseases have been influenced by public health mitigation measures during the COVID-19 pandemic, with stringent measures resulting in low incidence of various infectious diseases. Owing to the prevalence of SARS-CoV-2 infections and subsequent immunity development, decreasing virulence of SARS-CoV-2, and worldwide immunizations against SARS-CoV-2 in children aged >6 months, mitigation measures are lifted country-by-country. Consequently, immunity debt to respiratory viruses other than SARS-CoV-2 contributes to the “off-season,” “see-saw,” and “upsurge” patterns of infection in children. Apart from SARS-CoV-2 persistence, coexistence of other viruses or bacteria may lead to future twindemics or tripledemics. Therefore, maintaining hand hygiene and immunization policies against a variety of pathogens is necessary to alleviate the ongoing impacts of infectious diseases on children’s health.

## Author contributions

M-C Y and Y-T S contributed to conception and design of the study. M-C Y and Y-T S organized the database. M-C Y wrote the first draft of the manuscript. Y-T S, P-H C, C-C T, and T-I L wrote sections of the manuscript. J-R W coordinated and supervised data collection, collected data, and reviewed and revised the manuscript.All authors contributed to the article and approved the submitted version.
